# Secular trends: a ten-year comparison of the amount and type of physical activity and inactivity of random samples of adolescents in the Czech Republic

**DOI:** 10.1186/1471-2458-11-731

**Published:** 2011-09-26

**Authors:** Dagmar Sigmundová, Walid El Ansari, Erik Sigmund, Karel Frömel

**Affiliations:** 1 Center for Kinanthropology Research, Institute of Active Lifestyle, Faculty of Physical Culture, Palacky University in Olomouc, Tr. Miru 115, 77111 Olomouc, Czech Republic; 2 Faculty of Applied Sciences, University of Gloucestershire, Oxstalls Campus, Oxstalls Lane, Gloucester GL2 9HW, UK

## Abstract

**Background:**

An optimal level of physical activity (PA) in adolescence influences the level of PA in adulthood. Although PA declines with age have been demonstrated repeatedly, few studies have been carried out on secular trends. The present study assessed levels, types and secular trends of PA and sedentary behaviour of a sample of adolescents in the Czech Republic.

**Methods:**

The study comprised two cross-sectional cohorts of adolescents ten years apart. The analysis compared data collected through a week-long monitoring of adolescents' PA in 1998-2000 and 2008-2010. Adolescents wore either Yamax SW-701 or Omron HJ-105 pedometer continuously for 7 days (at least 10 hours per day) excluding sleeping, hygiene and bathing. They also recorded their number of steps per day, the type and duration of PA and sedentary behaviour (in minutes) on record sheets. In total, 902 adolescents (410 boys; 492 girls) aged 14-18 were eligible for analysis.

**Results:**

Overweight and obesity in Czech adolescents participating in this study increased from 5.5% (older cohort, 1998-2000) to 10.4% (younger cohort, 2008-2010). There were no inter-cohort significant changes in the total amount of sedentary behaviour in boys. However in girls, on weekdays, there was a significant increase in the total duration of sedentary behaviour of the younger cohort (2008-2010) compared with the older one (1998-2000). Studying and screen time (television and computer) were among the main sedentary behaviours in Czech adolescents. The types of sedentary behaviour also changed: watching TV (1998-2000) was replaced by time spent on computers (2008-2010).

The Czech health-related criterion (achieving 11,000 steps per day) decreased only in boys from 68% (1998-2000) to 55% (2008-2010). Across both genders, 55%-75% of Czech adolescents met the health-related criterion of recommended steps per day, however less participants in the younger cohort (2008-2010) met this criterion than in the older cohort (1998-2000) ten years ago. Adolescents' PA levels for the monitored periods of 1998-2000 and 2008-2010 suggest a secular decrease in the weekly number of steps achieved by adolescent boys and girls.

**Conclusion:**

In the younger cohort (2008-2010), every tenth adolescent was either overweight or obese; roughly twice the rate when compared to the older cohort (1998-2000). Sedentary behaviour seems relatively stable across the two cohorts as the increased time that the younger cohort (2008-2010) spent on computers is compensated with an equally decreased time spent watching TV or studying. Across both cohorts about half to three quarters of the adolescents met the health-related criterion for achieved number of steps. The findings show a secular decrease in PA amongst adolescents. The significant interaction effects (cohort × age; and cohort × gender) that this study found suggested that secular trends in PA differ by age and gender.

## Background

Childhood and adolescence are key periods regarding the importance of physical activity (PA) [1] because the level of PA in adolescence influences PA in adulthood [2-4]. Regular PA benefits adolescents in developing their physical, mental and social assets [5]. The recommended level of PA for children and youth (aged 5-17) is to accumulate ≥ 60 minutes of moderate to vigorous intensity PA daily [6,7]. Based on the daily step counts, the recommended minimal amount of PA (number of daily steps) for Czech youth (14-18 years old) is 11,000 and 9,000 steps per day for boys and girls respectively [8]. Sufficient PA has positive effects on self-perception, reduces the risks of cardio-metabolic diseases, and contributes to the prevention of excessive body fat and insulin resistance in adolescents [9-11].

The insufficient level and constant decrease of PA with age support the importance of monitoring PA and its effects in childhood. A significant decrease in PA occurs mainly during adolescence (after 14 years of age), together with a simultaneous increase in sedentary behaviour, most likely due to an excessive use of computers [12-15]. Although PA of adolescents has attracted global debate regarding its characteristics (e.g. recommended levels, influence of organized PA on total PA, sedentary behaviour, age-related decline) [4,9,12,16], studies have usually focused only on assessing the levels of PA, without detailed information about the specific types of adolescents' PA [17]. The different types of PA and sedentary behaviour are important for understanding active and sedentary behaviours, and for effective design, planning, and support of interventions which aim to promote physically active lifestyles.

PA levels in children and adults have traditionally been measured by questionnaires, with attending advantages and limitations [18]. There have been calls, however, for the need for objective monitoring to be employed in order to precisely assess PA levels [18]. Combining several measurement approaches (i.e. objective and subjective measurement) can provide more holistic information about PA [19]. For instance, pedometers are objective, simple, inexpensive and feasible motion sensors for assessing PA [19-21], and have been recommended for assessing and supporting PA in children and adolescents [20,21]. Pedometers correlate strongly with accelerometers and are a simple and inexpensive valid option for assessing PA in research and practice [19].

The post-communist block countries (e.g. the Czech Republic) appear to have a tendency to replicate the 'negative' health trends that had been previously witnessed in economically developed Western countries: a decrease in PA and an increase of overweight and obesity [22]. Indeed, Central and Eastern European countries could learn from such 'negative' Western European and global experiences [23]. However, the monitoring of secular trends of PA is rarely undertaken, probably due to the time demands required for longitudinal monitoring [13,24]. Unsurprisingly, very few studies of sedentary behaviour alongside PA have been undertaken in Eastern or Central Europe [25]. The current study bridges this gap and examines both PA and sedentary behaviour of two cohorts of Czech adolescents ten years apart: it measured PA levels, its types, and secular trends; and also measured the related duration of sedentary behaviour, its types and secular trends. This information is critical for future programs aiming to enhance PA in youth and young adults in the Czech Republic and further afield in other Central and Eastern Europe nations that are in transition.

### Aim of the study

This study assessed the levels and secular trends of PA and sedentary behaviour of adolescents in the Czech Republic. The main aim was to explore secular trends from 1998-2000 to 2008-2010 of pedometer-determined PA, and also sedentary behaviour of these adolescents. Random samples of adolescents (aged 14-18 years) of two cohorts (older cohort - monitored during 1998-2000; younger cohort - monitored during 2008-2010) completed a 7-day PA monitoring using pedometers. The five specific objectives were to:

• For each cohort, describe the proportion of adolescents by their BMI categories (based on self-reported height and weight and international cut-off points);

• For each cohort, describe the proportions of adolescents meeting the health criterion of PA (achieving 9,000 or 11,000 steps per day for girls and boys respectively);

• Asses any secular trends in pedometer-monitored number of steps achieved during the whole week, weekdays and weekends by gender and by younger (14-<16 years) and older (16-18 years) adolescents;

• Asses any secular trends in self-reported types and duration of PA achieved during the whole week, weekdays and weekends by gender and by younger (14-<16 years) and older (16-18 years) adolescents; and,

• Asses any secular trends in self-reported types and duration of sedentary behaviour during the whole week, weekdays and weekends by gender and by younger (14-<16 years) and older (16-18 years) adolescents.

## Methods

### Ethics and procedures

This study was undertaken in the Czech Republic after approval by the Institutional Research Ethics Committee at Palacky University. Participation was voluntary; participants received no incentives and participants' guardian/s could withdraw their children from the study if they wished. Adolescents and their parents were provided with information about the aims, objectives and methods of the study before the start of PA monitoring. Data were anonymous and confidential and data protection was observed at all times. Each participant's guardian signed an informed consent for inclusion in the study.

### Participants

A list of all high schools in the Czech Republic was compiled. From this list 51 schools were randomly selected and invited to participate in the study. Only 4 schools declined the invitation to participate in this study, generating a response rate of 92%. If in agreement, one or two classes were randomly selected from the given school, and all students from the selected classes who provided a guardian's signed informed consent were recruited to the study (regardless of ethnicity, socioeconomic status, parents' education etc.). After the completion of this random sampling process, in addition, about 15 high schools that were not initially selected in the random sampling also wished to participate in the study. These schools were also included in the sample and followed the same random selection of one or two classes from each school as described above.

The study employed the same methods for the long term monitoring of PA of two cohorts (1998-2000 and 2008-2010) of Czech adolescents who were monitored during a typical habitual week (one that is without holiday or any unusual events). Monitoring was carried out during any of the months of September, October, November, March, April, May and June. From the two cohorts, all adolescents aged 14-18 who completed the 7-day pedometer-monitored PA in their respective cohort were selected. Then, for an in-depth understanding of the PA and sedentary behaviour of this age bracket, these adolescents were further categorized into two age groups: younger adolescents (aged 14 to <16 years); and older adolescents (aged 16-18 years).

In total 1,573 adolescents were invited to participate in the study (604 from 1998-2000 cohort; and 969 from 2008-2010 cohort). The response rate across both cohorts (those who were invited and actually participated) was 94% (1,479 adolescents). However, across both cohorts 39% (n = 577) of participants provided incomplete or incorrect data (e.g. missing weight, height, age, or mean daily steps count of >30,000 or <1,000 [26]), and in line with others [26], were excluded from the analysis. Hence the final data for analysis comprised 902 adolescents (620 from 2008-2010 cohort; and 382 from 1998-2000 cohort). Table 1. depicts the data eligible for analysis by gender, age groups (younger or older adolescents), cohort, age, BMI categories (according to international cut off points [27-29]), and by whether or not the adolescents achieved daily step counts that met the health recommendations.

**Table 1:** Sample characteristics: adolescents eligible for analysis by BMI and by meeting health recommendations^†^

	Older Cohort (1998-2000)		Younger Cohort (2008-2010)		
	
	n	%	n	%	P value
**Whole sample**					
Boys	201	52.6	209	40.2	<0.01
Girls	181	47.4	311	59.8	<0.01
**Younger adolescents (14 to < 16 years)**					
Boys	65	44.5	86	53.1	0.13
Girls	81	55.5	76	46.9	0.13
**Older adolescents (16 to 18 years)**					
Boys	136	57.6	123	34.4	<0.01
Girls	100	42.4	235	65.6	<0.01
BMI according to cut-off points*					
Normal weight	361	94.5	466	89.6	<0.01
Overweight	20	5.2	44	8.5	0.06
Obese	1	0.3	10	1.9	0.02
**Meet health recommendations for achieved daily step counts**^**†**^					
Boys	136	67.7	114	54.5	<0.01
Girls	136	75.1	230	74.0	0.73

n: sample size; statistical significance based on test for difference between proportions; * in accordance with international recommended cut off points [27-29]; ^†^Czech recommendations for health-enhancing daily number of steps achieved (9,000 or 11,000 for girls and boys respectively) [8]

### Assessment of physical activity and sedentary behaviour

The week-long PA monitoring comprised continuous all-day monitoring using the pedometer, and in addition, the completion of individual chart sheets (to record the data from the pedometer and to provide more detailed information about the type and duration of PA and sedentary behaviour).

Adolescents wore either the Yamax SW-701 or Omron HJ-105 pedometers on either the left or right side on the hip continuously for 7 days for at least 10 hours per day (excluding sleeping, hygiene and bathing). Participants chose whether to wear the pedometer on the left or right side, as previous research showed that pedometers did not significantly differ in their estimates depending on the side of the body they were worn on [30,31]. Adolescents were informed to wear the pedometer for the whole day (i.e. put on the pedometer in the morning and remove it before sleeping). Pedometers were not worn during water based activities, but the time spent performing water based PA was recorded into the chart sheets (see below). Both the Yamax and Omron pedometers have been tested (in terms of number of steps) against direct observation (actual steps tallied with a hand counter) at different speeds: both models did not significantly overestimate or underestimate the number of steps up to speeds of 107 m/min [31]. Both the Yamax and Omron models have demonstrated high intra-model reliability [32] and accuracy for step counting at speeds of 80 m/min and above, where both provided mean values that were within ±1% of the actual steps [31].

In addition, information regarding self-reported PA and sedentary behaviour in minutes for each day was also collected: adolescents recorded the type and duration in minutes of any performed PA (e.g. walking, running, work-related physical activity, fitness, sport games as football, hockey, volleyball, etc.); and of any sedentary behaviour (e.g. sedentary time spent on personal computers, watching TV, studying, at a restaurant, etc.). Each adolescent recorded this information into an individual chart sheet (adapted from published studies [33,34] and slightly modified to better fit the Czech context and culture [35]). Adolescents entered this information during the day and also in the evening, but, in agreement with others [8], durations of PA or sedentary behaviour lasting less than 10 minutes were not entered into the chart sheets.

### Statistical analysis

This study was analyzed across two cross-sectional cohorts carried out in 1998-2000 and 2008-2010. Statistical analysis was undertaken using STATISTICA v.8 and SPSS v.19. For the pedometer and self-reported data, multivariate ANOVA (MANOVA) test (2 × 2 × 2) with related Fisher LSD post-hoc test computed any significant differences between the numbers of steps achieved. In the 2 × 2 × 2 MANOVA, the number of steps achieved (whole week or weekdays days or weekend) was the dependant variable. The three independent variables comprised age (younger adolescent 14-<16 years, older adolescent 16-18 years), cohort (younger cohort 2008-2010, older cohort 1998-2000) and gender (boys, girls).

Association of seasonal variation with weekly number of steps achieved was tested using one-way ANOVA test. Differences between the two cohorts in terms of sample characteristics, BMI and meeting health recommendations were based on tests for difference between proportions.

## Results

### Sample characteristics and proportion of adolescents by BMI and by meeting health recommendations for number of achieved daily steps

*Whole sample: *participants' mean age in the older cohort (1998-2000) was 16.16 ± 0.89 years and in the younger cohort (2008-2010) was 15.84 ± 0.79 years. In relation to BMI, when the younger cohort was compared to the older cohort, there were no significant secular changes in the proportion of adolescents who were overweight, but there was a significant increase in the proportion of obese adolescents. As regards meeting the health recommendations for the number of achieved daily steps, there was a significant decrease in the proportion of boys who met the recommendations when the younger cohort was compared to the older cohort. However, there were no similar significant inter-cohort differences in the proportion of girls who met the recommendations (Table 1).

### Secular trends: pedometer-monitored number of steps by gender, cohort and by younger (14-<16 years) and older (16-18 years) adolescents

Analysis of variance was used to assess whether seasonal variation was associated with the number of steps achieved. There were no differences in weekly number of steps achieved across the different seasons for the whole sample (n = 902) (F = 1.50; p = 0.22) and also for each cohort individually [younger cohort (p = 0.07) and older cohort] (p = 0.11) (data not presented).

Table 2 shows the MANOVA findings. In addition to the main effects, there were several significant interactions between cohort and gender and between cohort and age (as regards the number of daily steps achieved).

**Table 2:** F-values of differences in number of steps achieved during the week, weekdays and weekends by cohort, gender and age

Variable/F_factor_	**F**_**cohort**_	**F**_**gender**_	**F**_**age**_	**F**_**cohort × gender**_	**F**_**cohort × age**_	**F**_**gender × age**_	**F**_**cohort × gender × age**_
**Whole sample**							
Week	26.65***	24.33***	6.78***	2.79	13.03***	1.47	1.41
Weekdays	45.78***	23.92***	9.82**	4.00*	16.96***	0.68	0.52
Weekend	0.002	9.56**	0.286	0.13	1.415	2.28	2.73

Based on multivariate analysis of variance; *p < 0.05; **p < 0.01; ***p < 0.001

**Figure 1:**
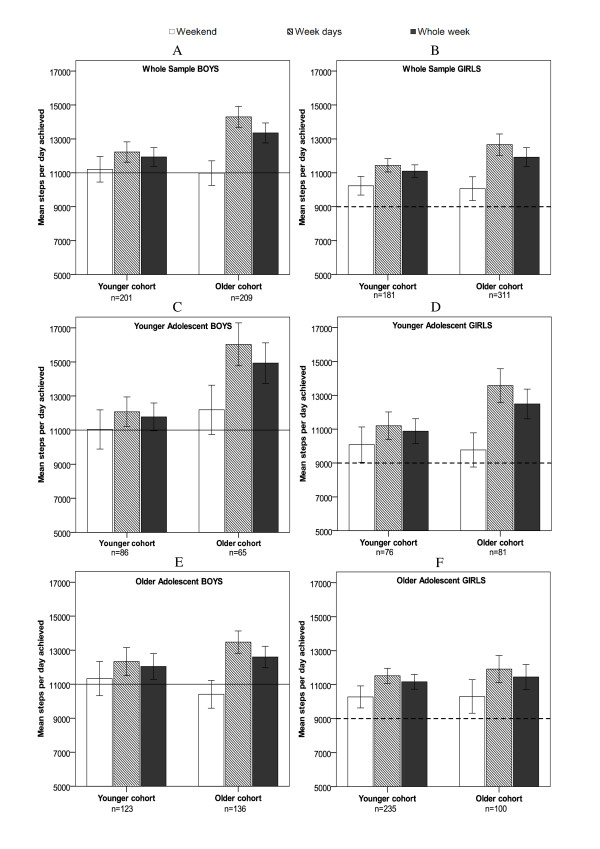
**Cohort differences by gender: daily steps achieved during the whole week, weekdays and at weekends ***Note*: --- recommendation (girls) 9,000 steps per day; -recommendation (boys) 11,000 steps per day; Error bars: 95% CI; older cohort (1998-2000); younger cohort (2008-2010); younger adolescent (14 to <16 years), older adolescent (16 to <18 years)

The comparison of the pedometer-monitored amount of PA for the whole sample of each cohort indicated several differences (Figures 1A and 1B). For the *whole week *(7-day monitoring), the interaction between cohort and age (Table 2) was significant (*p *< 0.001), with greater decreases (of the achieved weekly number of steps) in younger adolescents (14-<16 years) than in the older adolescents (16-18 years) when the younger cohort was compared to the older cohort. The comparison of the younger adolescents within the two cohorts (14 to <16 years, Figures 1C and 1D) shows that for the *whole week*, there was a significant decrease (post hoc LSD test) in the achieved number of steps of the younger cohort when compared with the older cohort for both genders (boys *p *< 0.0001; girls *p *= 0.008).

During *weekdays *(5-day monitoring), the MANOVA (Table 2) suggested similar interaction effects. The interaction between cohort and age was significant (*p *< 0.001), with greater decreases (of achieved weekly number of steps) in younger adolescents (14-<16 years) than in the older adolescents (16-18) when the younger cohort was compared to the older cohort. In addition, there was significant interaction between cohort and gender (*p *< 0.05) with greater decreases (of achieved weekly number of steps) in boys than in girls when the younger cohort was compared to the older cohort.

The post hoc LSD test indicated that in younger adolescents (14 to <16 years, Figures 1C and 1D) there were significant decreases in the step counts achieved during weekdays by the younger cohort, when compared to the older cohort for both genders (boys *p *< 0.0001; girls *p *< 0.001). In both genders, the younger adolescents of the older cohort (1998-2000) performed higher PA levels than their counterparts of the younger cohort (2008-2010) during the whole week and during working days.

In older adolescents (16 to <18 years, Figures 1E and 1F) during weekdays, there were significant decreases in the number of steps achieved by the younger cohort when compared to the older cohort in boys but not in girls (boys *p *= 0.02; girls *p *= 0.41).

However, during the *weekend *(2-day monitoring), there were no inter-cohort differences in the number of steps achieved (F = 0.002, *p *= 0.97), and also no interaction effects between cohort and other variables.

### Secular trends: duration and type of PA by gender, cohort and by younger and older adolescents

Table 3 depicts the secular differences in the self-reported type and duration of PA by gender, cohort and by younger (14-<16 years) and older (16-18 years) adolescent groups.

**Table 3:** F-values of differences in duration and types of PA by cohort, gender and age

**Variable/F**_**factor**_	**F**_**cohort**_	**F**_**gender**_	**F**_**age**_	**F**_**cohort × gender**_	**F**_**cohort × age**_	**F**_**gender × age**_	**F**_**cohort × gender × age**_
**Comparison by week segment (Duration as min/day)**							
Whole week	11.59***	1.65	6.34**	0.45	2.26	0.57	0.12
Weekdays	12.56***	1.88	10.88**	0.20	1.49	0.51	1.64
Weekend	0.37	1.04	4.78*	0.26	0.22	2.91	0.19
**Comparison by type (Duration as min/week)**							
Walking	13.76***	22.16***	0.03	0.73	1.55	0.21	1.27
Aerobic exercise	7.56**	0.003	7.41**	0.89	0.92	1.33	0.01
Games	0.10	42.97***	3.22	10.43**	3.22	3.99*	7.64**
Household	2.80	1.95	0.03	1.65	0.13	3.34	0.62
Other PA	0.73	10.13**	7.31**	0.03	0.01	0.27	2.74

Based on multivariate analysis of variance; *p < 0.05; **p < 0.01; ***p < 0.001

For the *whole week*, there were significant decreases in the duration of PA of the younger cohort when compared to the older cohort (F = 19.59, *p *< 0.001), where the older cohort (1998-2000) showed higher PA levels. The findings also indicated that younger adolescents reported significantly more PA than older adolescents (F = 6.34, *p *< 0.01). During *weekdays *(5-day monitoring), MANOVA (Table 3) suggested similar 'cohort' differences as there were significant decreases in the duration of PA of the younger cohort when compared to the older cohort (F = 12.56, *p *< 0.001). Similarly, younger adolescents reported significantly more PA than older adolescents (F = 10.88, *p *<0.01). During the *weekend *(2-day monitoring), there were only significant differences between younger and older adolescents (F = 4.78, *p *< 0.05) where younger adolescents (14 to < 16 years) reported more time spent in PA. Besides the significant main effects, there were no significant interactions between cohort, gender or age.

**Figure 2:**
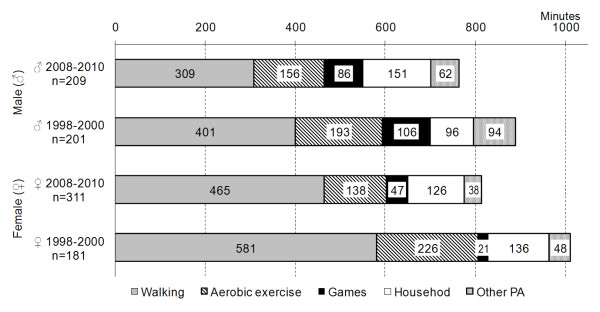
Structure of self-reported physical activity of adolescents (average value in minutes per week)

However, as regards the type of self-reported PA (Table 3), there were several significant interactions in the time spent playing games. For this activity, the interaction between cohort and gender was significant (*p *< 0.01), with increase of reported time spent playing games in girls (from 21 minutes to 47 minutes) and decreased time in boys (about 20 minutes, Figure 2), when comparing the younger cohort (2008-2010) to the older cohort (1998-2000). In addition, for this type of PA (time spent playing games), there was significant interaction effects between cohort, gender and age (*p *< 0.01). The post-hoc LSD test suggested that: 1) there were differences between cohorts in younger adolescent boys (*p *< 0.01); 2) there were gender differences in the older cohort for younger (*p *< 0.001) and older adolescents (*p *< 0.001); and, 3) there were age group differences in boys from older cohort (*p *< 0.001). In addition to these significant interactions, MANOVA (Table 3) also exhibited several significant main effects.

There were significant decreases in the time spent walking (F = 13.76, *p *< 0.001) and time spent in aerobic exercise (F = 7.56, *p *< 0.01) of the younger cohort (1998-2000) when compared to the older cohort (2008-2010). However, walking was a prevalent form of PA in both cohorts.

The proportional changes in total duration of self-reported PA between the two cohorts (Figure 2) were: for boys, the younger cohort generally exhibited less self-reported PA (by -9% to -18%) than the older cohort; for girls, generally, the younger cohort showed less self-reported PA (by -11% to -26%) than the older cohort.

### Secular trends: duration and type of sedentary behaviour by gender, cohort and by younger (14-<16 years) and older (16-18 years) adolescents

Table 4 depicts the secular differences in self-reported type and duration of sedentary behaviour by gender, cohort and by younger (14-<16 years) and older (16-18 years) adolescents. For the *weekdays*, there were significant interactions between cohort and gender (*p *< 0.05), with increase of the duration of sedentary behaviour in girls and a decrease in boys when the younger cohort is compared to the older cohort.

**Table 4:** F-values of differences in duration and types of sedentary behaviour by cohort, gender and age

**Variable/F**_**factor**_	**F**_**cohort**_	**F**_**gender**_	**F**_**age**_	**F**_**cohort × gender**_	**F**_**cohort × age**_	**F**_**gender × age**_	**F**_**cohort × gender × age**_
**Comparison by week segment (Duration as min/day)**							
Whole week	1.18	2.50	7.49**	2.22	2.57	2.38	0.01
Weekdays	2.04	6.94**	8.66**	4.39*	3.50	4.85*	0.10
Weekend	0.02	2.43	1.67	0.26	0.16	0.37	0.22
**Comparison by type (Duration as min/week)**							
Watching TV	29.28***	7.86**	3.23	3.88*	0.44	0.26	1.22
PC	51.75***	39.37***	0.76	0.46	0.26	0.05	1.86
Studying	35.70***	35.13***	7.14**	2.08	2.16	2.44	0.86
School	0.65	12.09***	16.81***	4.57*	1.30	2.16	3.78
Other sedentary behaviour	0.70	13.89***	0.20	0.13	11.14***	1.05	1.09

Based on multivariate analysis of variance; *p < 0.05; **p < 0.01; ***p < 0.001

During the *whole week *and *weekend*, there are no significant differences between cohorts.

MANOVA also suggested that there were significant interactions (*p *< 0.05) between cohort and gender in time spent watching TV, with greater decrease of time watching TV in boys than in girls, when comparing the younger cohort (2008-2010) to the older cohort (1998-2000). Similarly, there were significant interactions (*p *< 0.05) between cohort and gender in time spent sedentary at school, with decreasing time in boys and increasing time in girls, when comparing the younger cohort to the older one. There was also a significant interaction between cohort and age (*p *< 0.001) as regards other sedentary behaviour, (decreases in younger adolescents and increases in older adolescents), when the younger cohort is compared to the older cohort.

**Figure 3:**
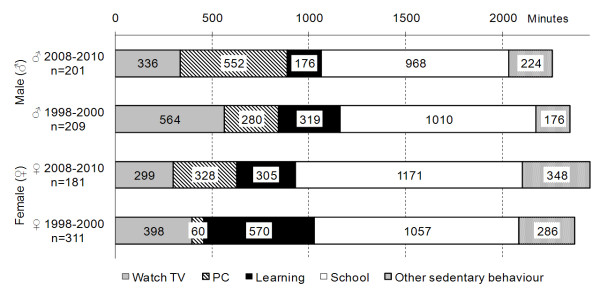
Structure of self-reported sedentary behaviour of adolescents (average value in minutes per week)

Apart from these significant interactions, MANOVA (Table 4) exhibited several significant main effects. There were significant increases in the time spent in front of PC (F = 51.75, *p *< 0.001), and significant decreases in time spent studying (F = 35.70, *p *< 0.001) of the younger cohort (2008-2010) when compared to the older cohort (1998-2000). The absolute values in self-reported sedentary behaviours are presented in Figure 3. The longest time adolescents spent sedentary was at school. Boys of both cohorts spent >14 hours per week watching TV or on computers; while girls' screen time in the older cohort was 458 minutes per week, increasing to 627 minutes per week in the younger cohort.

The proportional changes in total self-reported sedentary behaviour between the two cohorts were: for boys, the younger cohort (2008-2010) generally exhibited less self-reported sedentary behaviour (by -5% to -18%) than the older cohort (1998-2000). For girls (whole sample and older adolescents), the younger cohort (2008-2010) showed more self-reported PA than the older cohort (1998-2000).

## Discussion

A sufficient level of PA in adolescence is important for positive development of PA in adulthood [2-4,36]. Authors have repeatedly cautioned that significant decreases of PA appear mainly in adolescence [12-15]. However, Central and Eastern Europe seem to be going through similar negative health trends previously witnessed in Western countries despite the fact that these nations in transition could avoid such trends [23]. Hence, the current study assessed the secular trends in the total amount of PA (achieved number of steps) as well as the duration and types of PA and sedentary behaviours of both genders of two independent random samples of Czech adolescents across two cohorts ten years apart. Such research can provide indispensable evidence for future interventions aimed at the enhancement of PA in youth and young adults. In addition, the current study response rate (94%) is higher than in previous studies investigating secular trends [24].

In relation to the first objective, in these samples of adolescents from the Czech Republic, 10.4% and 5.5% were either overweight or obese in the younger cohort (2008-2010) and older cohort (1998-2000) respectively. Globally, the development of overweight and obesity is serious: for instance in the USA there was a 48% occurrence (of either overweight or obesity) in children and adolescents aged 10 to 17 years old [37]. In contrast, the HELENA study (9 countries in Europe) reported that 23% of adolescents were either overweight or obese [9]. Indeed, the progression of the obesity epidemic seems evident across Europe. In Switzerland, obesity in children increased from 4% (1960) to 18% (2003). In the UK, the proportion of obese children increased from 8% to 20% between 1974 and 2003. In Spain, the number of obese children doubled between 1985 and 2002 [22]. One of the plausible explanations for such increases in obesity observed in Europe and the USA, based on theoretical and empirical examinations, is the decline in PA [22,38,39]. In the current study's Czech sample, there was a minor increase (about 5%) in the proportion of overweight or obese adolescents across the 10-year period. Although the Czech sample of the current study showed more 'favourable' overweight or obesity levels than US adolescents [37] and European adolescents [9], the actual estimate of either overweight or obese Czech adolescents could be higher than this study's findings might indicate as the more physically active proportion of the population and particularly those with normal weight are usually more likely to consent to and complete studies of this nature [40].

In terms of the second objective, Vincent and Pangrazi [41] recommended achieving 11,000 and 13,000 daily steps respectively for girls and boys aged 6-12 years as a health criterion. Further, for the same age group, Tudor-Locke et al. [42] suggested 12,000 and 15,000 steps/day for girls and boys respectively. In the Czech Republic, the recommendation for adolescents (aged 14-18 years) is 9,000 and 11,000 daily steps for girls and boys respectively [8]. In the current study, over a 10-year period, the proportion of Czech boys who met the recommendation of 11,000 daily steps declined from 68% (older cohort, 1998-2000) to 55% (younger cohort, 2008-2010). In contrast, girls did not show a similar significant decline, whereby for both cohorts, approximately 74-75% of girls met the recommendation of 9,000 daily steps. Meeting this PA criterion seems to be appropriate to prevent increases of overweight and obesity [9]. This apparently high and stable proportion of Czech adolescent girls who achieved the PA recommendation is encouraging.

In relation to the third objective, the study assessed any secular changes in pedometer-monitored number of steps across two cohorts of adolescents 10-years apart. the decline of PA in connection with age is well documented [12,13], however, research on secular changes of PA have frequently not been undertaken (long time demands required for such studies) [13,24]. Indeed, only few such studies have been carried out in Eastern or Central Europe [25]. An important methodological finding is the significant interaction effects (cohort × age; and cohort × gender) that the current study found: this suggested that inquires into PA would need to analyse the number of steps achieved by cohort, age and gender. Surprisingly, other investigations of secular trends of adolescents' PA in Australia, Sweden and Spain did not report that any analysis was carried out in order to explore any such interactions [24,43-45].

The current study showed that for this sample of adolescent girls and boys in the Czech Republic, there was a secular negative trend (decrease) in terms of the achieved pedometer-monitored daily step counts. There were more decreases in the younger adolescents (14-<16 years) (for the whole week and also for weekdays). For the whole sample, there was greater decrease (of achieved weekly number of steps) in boys than in girls when the younger cohort was compared to the older cohort. However during the weekend there were no differences between the cohorts. In contrast, others [44] found no differences between Swedish adolescents' pedometer-monitored PA over four weekdays in 2000 and 2008, although a study of Swedish schoolchildren found a secular decrease from 2000 to 2006 in number of steps achieved during four consecutive weekdays [45]. Moreover, in Spanish adolescents, a positive secular change in health-related physical fitness (cardio-respiratory fitness and agility) was found over 5 years [43].

In summary, the current study's 'negative' finding is important and will require attention from policy makers: current Czech adolescents achieved a significantly less number of daily steps (notably during weekdays) than adolescents ten years earlier.

As for the fourth objective, the study examined any secular trends in the duration and types of self-reported PA across two cohorts. Generally, in this study, the total duration of self-reported PA in adolescents decreased 9%-18% in boys and 11%-26% in girls across the 10-year period. The adolescent population represents one of the most physically active subpopulations [17], however, researchers have usually assessed adolescents' PA without data about the specific types of the PA [17]. Bridging this gap, the current study monitored both the duration of PA as well its types. In this study's sample, walking accounted for the largest proportion of total PA, in agreement with PA studies carried out in Czech, Polish and Swedish adolescents [46,47], Filipino youth [48], or Canadian youth [49]. However, the current study findings also showed a secular decrease in time spent walking, which is contrary to Canadian adolescent girls, where it was the only activity where the prevalence did not decrease over time [49]. Walking is a prevalent form of PA in many countries, and it is a movement form that has great potential in global PA [17].

Other popular types of PA in girls are aerobic exercises e.g. cycling, in-line skating and running [47]. The current study observed a significant decrease in aerobic exercises when the younger cohort (2008-2010) was compared to the older cohort (1998-2000). PA promotion in adolescent girls might be enhanced by offering them their preferred activities (dance, aerobics, sport games) [50]. On the other hand, boys usually prefer sports games [47]. However, despite the popularity of sports games, in boys, the current study found a negative secular change in time spent playing games. Achieving sufficient PA in adolescence appeared to be most beneficial in enhancing adult PA e.g. adolescents' (boys) participation in ball games increased their participation in ball games in adulthood [1]. In summary, the current study found a negative secular trend in the duration of self-reported PA.

As regards the fifth objective, the study examined any secular trends in the duration and type of self-reported sedentary behaviours across the two cohorts. Sedentary behaviour in children such as watching TV can be a more crucial indicator of risk of obesity than PA behaviour [51]. In the current sample of Czech adolescents, for both genders, sedentary behaviours in both cohorts were not different. Increased time spent on computers seems to be compensated for by decreased time spent watching TV. This increased time spent on computers is consistent with the increase in households in the Czech Republic who own computer/s (from 17.9% of households in 2000 to 54.2% in 2009), and also consistent with the increase in households with internet connections (from 5.8% in 2001 to 49.2% in 2009) [52].

A more detailed analysis revealed that Czech adolescent boys spent about 2 hours daily watching TV or on computers, while girls spent about 1-1.5 hours. Although this amount of time might still seem acceptable, it is necessary to observe and/or possibly regulate it, as watching TV is a key factor that increases the risk of being overweight or obese [51,53]. The current study also found that a larger proportion of sedentary behaviour of Czech adolescents was during studying (4-6.5 hours/working day). Indeed watching TV, computer use and studying have been reported sedentary behaviours in adolescents in Hungary [25], Spain [54], and Finland [55]. However, it is encouraging that current (2008-2010) Czech adolescents self-reported less sedentary behaviour than their counterparts ten years ago (1998-2000).

This study has limitations. Due to the respondent burden, the sample comprised 902 adolescents. This study is not a population study, rather it is based on random samples. BMI was computed based on self-reported (not measured) height and weight which might be influenced by social desirability and sociability, and are not objective indicators. The study is also unable to estimate the extent of inaccurate completions of the record charts by the participants that might influence the durations of the reported PA and sedentary behaviours (e.g., missing/forgetting to record certain PA/sedentary behaviours; only roughly estimating the duration of PA/sedentary behaviours where the duration of reported PA/sedentary behaviours were not precisely measured by a watch). Two types of pedometers were used in this study. Even if both models demonstrated good reliability, this might cause some differences in the number of recorded steps. No steps were added for the water based PA, however these types of physical activities were not discounted: they were included and recorded in the charts in relation to the duration of self-reported PA. While the combination of objective and subjective methods of monitoring increases the validity of the monitoring, neither pedometers nor self-reports consider the intensity of performed PA. The use of pedometers to assess weekly PA could reflect reactivity as pedometers lack a blinded display, and furthermore, participants also registered the number of daily steps into the record charts: these facts suggested that pedometers could be 'semi-objective'. The recommendations for Czech adolescent were created before the year 2000, and new knowledge about PA and sedentary behaviour could contribute to new, more valid recommendations for Czech adolescents. Future PA monitoring would need to address these factors, and would benefit from using motion devices without display (e.g. Actigraph) to provide more precise objective estimates; recruiting more participants; and, implementing longer term monitoring e.g. four-week long comparative studies implemented in different school environments (sport schools, boarding schools, schools implementing special educational programs, e.g. "healthy schools"), or under different socio-demographic conditions.

## Conclusions

Levels of overweight or obesity were not highly prevalent in this sample of Czech adolescents. Only 10.4% and 5.5% of adolescents in the Czech Republic were either overweight or obese in the younger cohort and older cohort respectively. Most Czech adolescents met the health criterion for PA (notably in the older cohort). The proportion of girls who achieved the criterion of 9,000 steps per day was stable (did not change) across both cohorts. In boys, there was a slight decrease in the proportion of current adolescents (the younger cohort) who met the criterion in contrast to the older cohort of 1998-2000. In this Czech sample, there was a secular decrease in the amount (number of steps) of PA and also, with some exceptions, in the duration of self-reported PA across a ten year period. On the other hand, there were no identified secular changes in the time spent in sedentary behaviour in boys, although there was a slight secular increase of sedentary behaviour in girls across the same time period. There was an increase of time spent on computers, with a simultaneous decrease in time spent watching TV. The main items stated for sedentary behaviour were TV watching and computer use. Future intervention programs should focus on long-term changes in post-communist countries, and as the most prevalent form of PA in the Czech Republic is walking, therefore future health promotion efforts should build on this in order to encourage and expand the population base that is undertaking this type of PA. Interaction effects also showed that PA differed across cohorts by age and by gender, hence future studies need to consider such interaction effects.

## Competing interests

The authors declare that they have no competing interests.

## Authors' contributions

The ES and KF created the concept and design of the study. DS, ES and WEA undertook the data analysis. DS and WEA wrote this manuscript with the input of all the co-authors. All the authors approved the final version.

## Pre-publication history

The pre-publication history for this paper can be accessed here:

http://www.biomedcentral.com/1471-2458/11/731/prepub
